# Icariin as a Treatment Proposal in Mammalian Reproduction

**DOI:** 10.3390/ph17091104

**Published:** 2024-08-23

**Authors:** Manuel Sánchez-Gutiérrez, Aleli Julieta Izquierdo-Vega, Eduardo Osiris Madrigal-Santillán, Claudia Velázquez-González, Jeannett Alejandra Izquierdo-Vega

**Affiliations:** 1 Academic Area of Medicine, Institute of Health Sciences, Autonomous University of the State of Hidalgo, Ex-Hacienda la Concepción, Tilcuautla 42160, Mexicoaleli_izquierdo11168@uaeh.edu.mx (A.J.I.-V.); eduardo_madrigal3215@uaeh.edu.mx (E.O.M.-S.); 2 Academic Area of Pharmacy, Institute of Health Sciences, Autonomous University of the State of Hidalgo, Ex-Hacienda la Concepción, Tilcuautla 42160, Mexico; claudiav@uaeh.edu.mx

**Keywords:** icariin, male infertility, female infertility, reproductive

## Abstract

Icariin (ICA), one of the main active components of *Herba Epimedii*, is a natural prenylated flavonol glycoside that possesses a wide range of pharmacological effects, including antioxidant, antiosteoporotic, anti-aging, neuroprotective, immunomodulatory, antitumor, and aphrodisiac effects, and prevents numerous health disorders, such as cardiovascular diseases, osteoporosis, cancer, sexual dysfunction, menstrual disorders, neurodegenerative diseases, asthma, chronic inflammation, and diabetes. In the reproductive system, it has been observed that ICA may play a role in preserving fertility by regulating different signalling pathways, such as PI3K/AKT, which improves ovarian function, and ERα/Nrf2, which enhances testicular function and prevents ROS generation. In contrast, regulating the NF/kB signalling pathway causes anti-inflammatory effects, reducing spontaneous abortions. In this study, we review and examine the relevant literature on the therapeutic potential of ICA in reproduction, highlight the various mechanisms of action and limitations for the therapeutic applications of ICA, and summarise and highlight the existing preclinical research on its effects on male and female reproduction.

## 1. Introduction

**Figure 1:**
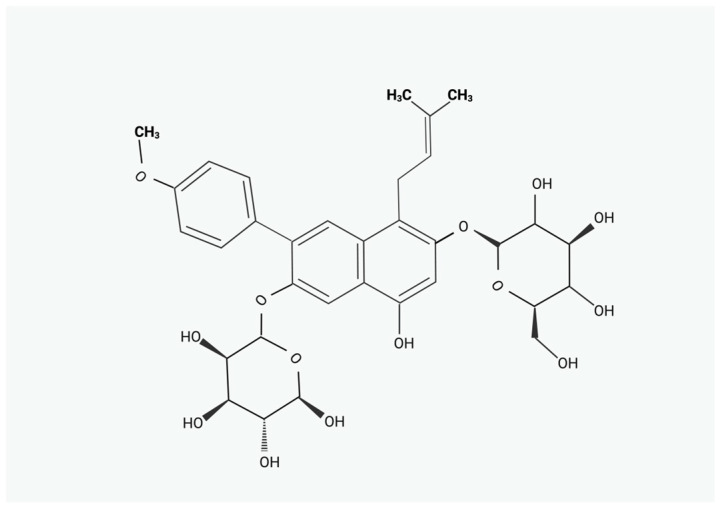
Chemical structure of ICA.

China, Korea, and Japan have used the dried leaf of the genus *Epimedium* L., also known as barrenwort, horny goat weed, Yin Yang Huo, and *Herba Epimedii*, as a traditional medicinal herb for more than two thousand years; approximately 62 species are currently known to exist in China [1]. Icariin (ICA), known as the 8-prenyl derivate of kaempferol 3,7-O-diglucoside, contains a prenyl group attached to carbon 8 of ring A of the flavonol glucoside. Its molecular weight is 676.67 g/mol, and its formula is C_33_H_40_O_15_ (Figure 1). With a range of biological functions, ICA is the primary active ingredient in *Epimedium*. *Epimedium* L. contains about 379 chemicals, most of which are prenyl flavonoids, terpenoids, lignans, and other constituents [2]. Pharmacokinetic studies carried out in rats have identified several metabolites of ICA in bodily fluids, including in the stomach, small intestine, bile, urine, and faeces, following a single dose of ICA. The estimated half-life of ICA after a single dose of (0.69 g/kg) is 3.149 h [3]. The metabolism starts in the small intestine and results in the formation of icariside II and icaritin. The transformation of icariside I through hydroxylation, demethylation, glucuronidation, and desugarisation has been found in 17 distinct metabolites [4]. Furthermore, in vitro tests have shown that ICA is metabolised through the human intestinal microbiota, generating different metabolites depending on the bacteria tested [5]. Studies have reported that ICA and its metabolites were found in the stomach, small intestine, bile, urine, and faeces of rats administered with ICA by gavage [6]. In the rats’ stomach, ICA was relatively stable, and only a small amount of icariside I and icariside II was detected [3]. A significant amount of icaritin and icariside II was detected in the small intestine. ICA and its main metabolites, icariside I and icariside II, were detected in the bile and urine in rats [7]. In addition, 11 metabolites were detected in faecal samples, and the main metabolites were icariside II, icaritin, and desmethylicaritin in rats [8].

ICA has been shown to have a wide range of therapeutic properties at different levels, such as in the cardiovascular [9], nervous [10], urinary [11], skeletal [12], immune [13], and reproductive systems [14]. Likewise, several properties of ICA have been recognised, including antioxidant, anti-inflammatory, immunoregulatory, antiproliferative, antithrombotic, anti-atherosclerotic, antihypertensive, hepatoprotective, neuroprotective, anti-ageing, and antidepressant properties [13,15,16]. Some studies have shown different mechanisms of action: it works as a phytoestrogen [17], a phosphodiesterase inhibitor [18], and a bone density-preserving agent [19]. Although the role of ICA as a phytoestrogen may seem to contrast its beneficial effects on reproduction, not all phytoestrogens alter male fertility or the hypothalamic–pituitary–testis axis [20]. Specifically, previous studies have indicated that the phytoestrogenic effect of ICA is tissue-selective [21], and its metabolites, such as icariside I, icariside II, and icaritin, have not yet demonstrated oestrogenic effects [22]. Therefore, the response to phytoestrogen may vary depending on the species, dose, and type of phytoestrogen. In addition, ICA presents anticancer activity, inhibiting the growth, proliferation, and migration of cancerous tumours. In addition, it can induce apoptosis in cancer cells and block the cell cycle. Its effectiveness has been demonstrated against different types of cancer, such as breast, colorectal, oesophageal, ovarian, and pancreatic tumours [23,24]. Furthermore, it improves the pathophysiological characteristics of Alzheimer’s disease [25].

## 2. Effects of ICA on Male Reproductive Function

**Figure 2:**
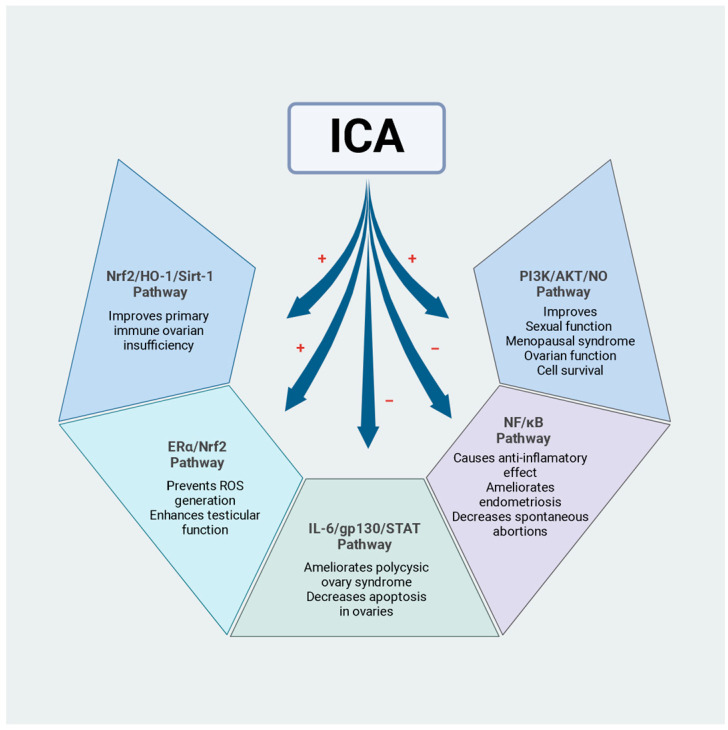
Regulation of cellular pathways by ICA. Effects on the reproductive system. “+” indicates activation, while “−” indicates inhibition.

Numerous studies on the effects of ICA on erectile dysfunction and male reproductive function have been published in recent years. Table 1 summarises these studies, demonstrating that ICA protects against oxidative damage and has beneficial effects on reproductive dysfunctions associated with diabetes or environmental pollutants. Modulation of cellular pathways involved in reproductive protection, mediated by ICA, is also shown in Figure 2.

The evaluation of the effects of ICA at the reproductive level on erectile function began almost 20 years ago. ICA was administered to castrated rats at doses of 1 and 5 mg/kg for 4 weeks. The results indicated an increase in the percentage of smooth muscle, intracavernous pressure during electrostimulation of the cavernous nerve, and the protein expression of neuronal nitric oxide synthase (nNOS) and inducible nitric oxide synthase (iNOS), indicating a potential therapeutic effect on erectile dysfunction [26]. ICA inhibited different isoforms of phosphodiesterase 5 (PDE5) in cavernosal smooth muscle cells and increased the cGMP levels compared to the control. Despite being less potent in inhibiting PDE5, ICA improved the cGMP levels in cavernosal smooth muscle cells more effectively than zaprinast [27].

There is also evidence that ICA inhibits other phosphodiesterases such as PDE4 and PDE5 [28]. Regarding other PDEs involved in reproduction, such as PDE3A, whose deficiency affects oogenesis [29], the effect of ICA on PDE3A has not yet been evaluated in experimental animals or clinical trials on male or female fertility. In this regard, in vitro studies show that other non-selective phosphodiesterase inhibitors improve human sperm motility and hyperactivation for in vitro fertilisation procedures [30]. Likewise, milrinone, a PDE3 inhibitor in human sperm, slightly increased the cAMP levels without affecting sperm functions [31]. Further studies are required to demonstrate the effect of ICA and the role of the PDEs involved in spermatogenesis or oogenesis.

The antioxidant effects of ICA have also been highlighted in an in vitro model. This study tested concentrations of 0.001, 0.01, and 0.1 µg/mL of ICA on human spermatozoa with oxidative damage induced by FeSO_4_/H_2_O_2_. The changes were evaluated using Raman microspectroscopy. Treatment with ICA protected the sperm from oxidative damage caused by FeSO_4_/H_2_O_2_ because ICA allowed the Raman fingerprint to be preserved, like in sperm cells without oxidative damage. Furthermore, ICA increased the activity of the sperm enzymes lactate dehydrogenase (LDH) and superoxide dismutase (SOD), compared to the FeSO_4_/H_2_O_2_-treated group [32].

In another study, assessed that ICA mouse-induced pluripotent stem cells (iPSCs) can differentiate themselves into spermatozoa in vitro. To create germ cells, pluripotent stem cells from mice were induced and cultivated. The techniques used were RT-PCR and Western blot. Within a specific range and in a concentration-dependent way, ICA can facilitate the in vitro transformation of mouse iPSCs into spermatozoa. Higher concentrations of icariin (100 μg/mL) showed a higher transformation efficiency compared to lower concentrations (0.1–10 μg/mL). Primitive germ cells obtained in vitro from mouse iPSCs especially express markers such as Oct-4 protein, C-kit protein, Mvh mRNA, Fragilis mRNA, and Stella mRNA, indicating the successful derivation of primordial germ cell-like cells. The obtained spermatozoa especially expressed VASA, SCP3, and γH2AX proteins. RT-PCR showed that the spermatozoa especially expressed Ddx4, Tp2, and Prm1 mRNAs. The results suggest that ICA can improve the transformation efficiency of mouse iPSCs into spermatozoa in vitro in a concentration-dependent manner [33].

### Mechanisms of Action of ICA in the Protection of Male Reproduction

The effect of ICA on male sexual function has been previously studied in an experimental mating model in mice. ICA doses of 50, 100, and 200 mg/kg were administered by gavage for 21 days. The evaluation methods used in this study included mating behaviour, measurement of genital indices, serum testosterone, NO levels, hypothalamic dopamine (DA) and 5-HT concentrations, and eNOS expression in penile tissues. Western blot analysis was performed to detect the expression of PI3K and p-AKT in penile tissues and investigate the mechanism of ICA in male sexual function. The effects of ICA on sexual behaviour and male sexual function were dose-dependent: the high-dose group showed the strongest impact, followed by the medium- and low-dose groups. ICA significantly improved sexual behaviour, increased the testicular and prostate indices, elevated the serum testosterone and NO levels, and improved the hypothalamic DA and 5-HT levels. It also positively regulated the expression of eNOS in penile tissues. ICA activated the PI3K/AKT pathway, which improved sexual function through the NO signalling pathway [34]. Moreover, the effect of ICA on reproduction has also been evaluated in animal models. In a study conducted in rats, doses of 50 mg/kg ICA, 100 mg/kg ICA, and 200 mg/kg ICA were evaluated for 35 consecutive days. A significant increase in the testosterone levels was observed for doses of 50 and 100 mg/kg. A 100 mg/kg dose of ICA increased the mRNA expression of genes in the rats’ testes, such as the benzodiazepine receptor, the steroidogenic acute regulatory protein (StAR), and the luteinizing hormone receptor (LHR). It also increased the activity of superoxide dismutase and decreased the level of lipid peroxidation in the testes. Thus, ICA may present benefits for male reproduction [35].

ICA has also been evaluated in animals with diabetes mellitus, where it improves reproductive dysfunction. In an experimental study, Sprague–Dawley rats with streptozotocin-induced diabetes and were fed a high-fat diet were administered with 80 mg/kg ICA for 6 weeks. ICA improved the number of epididymal sperm, increased sperm motility, and restored the serum levels of sex hormones (luteinizing hormone (LH), follicle-stimulating hormone (FSH), and testosterone). It also increased the expression of sirtuin-1 and hypoxia-inducible factor 1-alpha, positively regulated the expression of proliferating cell nuclear antigen (PCNA), and reduced testicular apoptosis in diabetic rats fed a high-fat diet. Furthermore, ICA treatment increased the number of spermatogonia, primary spermatocytes, and Sertoli cells by positively regulating sirtuin-1 activity (SIRT-1). Additionally, compared to metformin, it was more effective in preventing insulin resistance and anti-hypoxia, as evidenced by the positive regulation of hypoxia-inducible factor 1-α (HIF-1α) [36].

Furthermore, ICA is capable of improving testicular dysfunction caused by environmental pollutants. Researchers have previously studied the protective effect of ICA on exposing Leydig cells to di(2-ethylhexyl) phthalate (DEHP), one of the most common environmental endocrine disruptors. In the DEHP-exposed cells previously treated with ICA (0.2, 1, and 5 µg/mL), ICA reversed the adverse effects of DEHP on Leydig cells: it decreased the ROS levels, increased the mitochondrial membrane potential, and promoted testosterone production. It regulated the expression of transcription factor SF-1 and steroidogenic enzymes. In the same study, the protective effect of ICA was evaluated in an in vivo model in mice exposed to DEPH and ICA. While DEPH decreased the number of sperm cells in the epididymis and damaged the seminiferous tubules, 50–150 mg/kg of ICA decreased the effects caused by DEPH by preventing accumulation by increasing the epididymal sperm count [37].

Furthermore, the anti-ageing effects of ICA on male reproductive function have also been evaluated. In an experimental study in aged Sprague–Dawley rats given ICA at doses of 2 and 6 mg/kg for 4 months, the sperm concentration, testosterone levels, and histological evaluations were assessed after ICA treatment. ICA significantly ameliorated the age-related decline in testicular function and caused an increase in sperm count and viability, as well as an increase in the testosterone and oestradiol concentrations in the testicles. It also increased the number of Sertoli cells, improving their structure and activating the ERα/Nrf2 signalling pathway. The same study assessed the protective effects of ICA against D-galactose-induced injury using an in vitro model with TM4 cells through various assays, including intracellular ROS levels, protein expression level measurement, and Nrf2 signalling activation. The Nrf2 and oestrogen/ERa signalling pathways played a role in the protective effects of ICA against testicular dysfunction and Sertoli cell injury. Experiments evaluating Nrf2 expression levels and signalling activation revealed that ICA attenuated Sertoli cell damage in TM4 cells by activating the ERα/Nrf2 signalling pathway. ICA protected Sertoli cells by boosting Nrf2 and the downstream signalling molecules, such as haemeoxygenase-1 (HO-1) and NADPH: quinone oxidoreductase (NQO-1), promoting Nrf2 nuclear translocation and preventing ROS generation in D-galactose-stimulated TM4 cells. Furthermore, Nrf2 knockdown resulted in TM4 cell death, demonstrating the significance of Nrf2 for Sertoli cell function. These results imply that the ERa/Nrf2 pathway mediates the protective effects of ICA on Sertoli cell damage [38].

The protective effect of ICA against the toxic effects of nicotine in male mice has also been reported. Nicotine exposure at 0.75 mg/kg caused a significant decrease in the number of epididymal spermatozoa and the concentration of serum testosterone, in addition to oxidative damage in the testes. Animals were administered 75 mg/kg/day of ICA via gavage for 35 days. Testicular morphology, serum testosterone levels, antioxidant enzyme activity, and mRNA expression levels were measured after treatment. The results showed an increased sperm concentration, an increased serum testosterone concentration, and the positive regulation of the mRNA expression of antioxidant genes, counteracting nicotine-induced stress. The study suggests that ICA may be a therapeutic agent in cases of spermatozoa quality impairment due to smoking [39].

Recently, a study highlighted that ICA regulates the glycolytic pathways to improve the spermatogenesis deficiencies in obese mice caused by a high-fat diet. The mice were administered ICA at a dose of 80 mg/kg/day by gavage for 12 weeks. In the same study, the effect of ICA on palmitic acid-induced damage was evaluated using an in vitro model with TM4 cells. The analysis methods included semen quality, histological and immunohistochemical analyses, stained with H&E in the testes and epididymis, as well as Western blot and RT-PCR analyses. The study showed that, after treatment with ICA, the body weight and the proportion of abnormal spermatozoa were reduced in obese mice. The sperm count and the number of spermatogenic cells were also increased. The ICA treatment improved the histopathological changes observed in obese mice and decreased testicular apoptosis by downregulating Bax expression, upregulating Bcl2 and PNCA expression, and, in Sertoli cells, improving vimentin markers, WT1, and GATA4. Likewise, ICA improved cell viability and apoptosis and also reduced the lactate levels, restored the glycolytic processes, and highlighted the improvement in spermatogenesis under conditions of metabolic stress [40].

Finally, in an experimental study, researchers assessed the preventive impact of an ICA complex, including zinc, an important trace element for reproductive system function, on exercise-induced weariness and the reproductive system in Wistar rats. This work created an ICA–zinc compound, and its molecular formulas were determined using inductively coupled plasma atomic emission spectroscopy (ICP-AES). Using the experimental post-swimming anti-fatigue method and test indices such as the body weight, the testosterone levels, and the weight of the sexual organs, the experimental animals were given low and high dosages of the zinc complex (60 and 180 mg/kg of ICA–zinc, respectively) for three weeks. The ICA–zinc complex increased the exercise capacity in rats by prolonging their swimming time and significantly increasing their serum testosterone levels. Furthermore, the combination lowered the muscle glycogen stores, indicating improved glycogen usage effectiveness. In the treatment groups, the testicular weight somewhat increased; however, the ICA–zinc complex showed a more significant increase, suggesting the enhanced function of the reproductive system. Dose-dependent improvements in some markers associated with exercise fatigue and reproductive system glands were observed with the ICA–zinc complex [41].

**Table 1:** Summary of the main studies on the effects of ICA on male reproduction.

Type of Study	Model	Dose and Duration of Treatment	Main Findings	Refs.
In vivo	Male Wistar rats	1 and 5 mg/kg/4 weeks	ICA significantly increased the intracavernous pressure, the percent of smooth muscle, and the expression of neuronal and inducible nitric oxide synthase in castrated rats.	[26]
In vitro	Human spermatozoa	0.001, 0.01, 0.1 μg/mL/45 min	ICA protected human sperm cells from being damaged by FeSO_4_/hydrogen peroxide. Also, ICA increased the activity of the sperm enzymes lactate dehydrogenase and superoxide dismutase compared to sperm cells treated with FeSO_4_/hydrogen peroxide.	[32]
In vivo	Male Sprague–Dawley rats	45 mg/kg ICA, 110 mg/kg zinc 60 mg/kg ICA, 110 mg/kg zinc 180 mg/kg ICA, 110 mg/kg zinc	The ICA–zinc complex promoted development in male rats, improved their excitability, promoted their recovery from the fatigue state, and enhanced their anti-fatigue ability. The ICA–zinc complex increased the plasma testosterone concentration and the testicular weight, indicating improved function.	[41]
In vivo	ICR male mice	50, 100, and 200 mg/kg, 21 days	Increased serum testosterone and nitric oxide concentrations, hypothalamic dopamine and 5-hydroxy-tryptophan levels, and endothelial nitric oxide, phosphatidylinositol tallow alcohol 3-kinase, and phosphorylated protein kinase expression in penile tissues.	[34]
In vivo	Male Sprague–Dawley rats	50, 100, and 200 mg/kg, 35 days	100 mg/kg ICA significantly increased the epididymal sperm counts. 50 and 100 mg/kg ICA significantly increased the testosterone levels. 100 mg/kg ICA treatment also increased follicle-stimulating hormone receptor (FSHR) and claudin-11 mRNA expression in Sertoli cells.	[35]
In vivo	Male Sprague–Dawley rats	80 mg/kg, 42 days	ICA effectively attenuated male reproductive dysfunctions and spermatogenesis deficiencies induced by diabetes mellitus. ICA may ameliorate diabetes mellitus-induced spermatogenesis deficiency possibly through increasing proliferation and inhibiting intrinsic mitochondria-dependent apoptotic pathways of spermatogonia, primary spermatocytes, and Sertoli cells, respectively. ICA may be a potentially novel therapeutic agent for the protection and treatment of testicular damage in diabetes mellitus.	[36]
In vitro In vivo	Mouse Leydig cells ICR male mice	0.2, 1, and 5 µg/mL 3 h pre-treatment/12 h culture 50, 100, and 150 mg/kg, 28 days	ICA reversed the adverse effect of di(2-ethylhexyl) phthalate (DEHP) on Leydig cell proliferation, decreased the reactive oxygen species levels, and elevated the Δψm levels. Additionally, ICA promoted testosterone production and upregulated the expression of transcription factor (SF-1) and steroidogenic enzymes. ICA reversed the deleterious effects caused by DEHP on the epididymal sperm count and the seminiferous tubules.	[37]
In vivo In vitro	Male Sprague–Dawley rats Sertoli cell line TM4	2 and 6 mg/kg, 4 months 0.5 and 1 µM, 20 h	ICA significantly increased the testicular and epididymal weights and their indices in ageing rats. Furthermore, ICA increased sperm count and sperm viability. ICA protected against Sertoli cell injury due to age-related testicular dysfunction by upregulating the ERa/Nrf2 signalling pathway.	[38]
In vivo	Male Kunming mice	75 mg/kg, 35 days	ICA improved the reduction in sperm density, hormone levels, and antioxidant enzyme activity seen in nicotine-treated mice.	[39]
In vivo In vitro	Male C57BL/6J Sertoli cell line TM4	80 mg/kg, 12 weeks 1 µM ICA plus 0.4 mM palmitic acid, 24 h	ICA treatment improved the histopahological changes and decreased apoptosis in the testes of obese mice. It increased the expression of Bcl2, PCNA, WT1, GATA4, and vimentin. ICA improved the decreases in the lactate levels, the increases in pyruvate production, and the expression of HK2, PKM2, and LDHA induced by palmitic acid in TM4 cells.	[40]

## 3. Effects of ICA on Female Reproductive Function

There is evidence on ICA’s effects on female reproduction as well, even though the majority of studies thus far have concentrated on male reproduction. Table 2 summarises the studies that have demonstrated different beneficial effects on female fertility, reducing the frequency of miscarriages, ovarian function, and symptoms of polycystic ovary syndrome.

In one study, rat ovarian granulosa cells cultured in vitro and treated with different concentrations of hyperin and ICA were used. This study investigated the effects of hyperin and ICA on granulosa cells, focusing on cell proliferation, oestrogen and progesterone secretion, and CYP17 and CYP19 expression. Rat ovarian granulosa cells were cultured and subjected to varying concentrations of hyperin and ICA as part of the research methodology. The oestrogen levels were assessed by ELISA and the MTT test for cell proliferation. qRT-PCR was used to identify the mRNA expression of CYP17 and CYP19. Furthermore, the protein expression levels of CYP17 and CYP19 were evaluated by Western blotting. The results showed that hyperin (50 μg/L) and ICA (10 μg/L) increased cell proliferation, as well as oestrogen and progesterone secretion. These compounds also upregulated the expression of CYP17 and CYP19 in granulosa cells. These results suggest that hyperin and ICA promote oestrogen and progesterone biosynthesis by upregulating aromatases, potentially improving ovarian endocrine function and pregnancy outcomes [42].

Another study has analysed the effect of ICA on hydrogen peroxide treatment in mice embryos in vitro. The effect of 60 µM hydrogen peroxide was evaluated with different concentrations of ICA in the range of 10 to 80 µM in mice embryos. The methods used in this study included treating mouse embryos with hydrogen peroxide to induce oxidative damage, followed by ICA supplementation until the blastocyst stage. The ROS levels were detected using DCFH-DA, and the mitochondrial membrane potential (ΔΨm) was assessed by JC-1 staining. RT-PCR was used to observe the expression of zygotic gene activation (ZGA-3) and see how ICA affected gene activation during the development of preimplantation embryos. The hydrogen peroxide treatment elevated the ROS, which affected embryonic development, while the ICA treatment reversed these adverse effects by modulating mitochondrial activity and the mRNA expression of eIF-1A, a ZGA-3 marker gene. The study also highlighted the importance of controlling the ROS levels in preimplantation embryos and the potential of antioxidants such as ICA in promoting embryonic development by reducing the ROS levels. The most effective dose of ICA to reverse the adverse effects of hydrogen peroxide was 40 μM. The ICA treatment restored the mitochondrial membrane potential levels, indicating its role in improving ATP production and promoting embryonic development [43].

### Mechanisms of Action of ICA in the Protection of Female Reproduction in In Vivo Models

Another study has shown that ICA affects menopausal changes; the tissue-selective oestrogenic activities of ICA in oestrogen-sensitive tissues have been evaluated in vivo and in vitro. Treatment for 12 weeks with ICA at a dose of 500 mg/kg prevented deficiency-induced osteoporotic changes in the bone structure, bone mineral density, and trabecular column. It also showed oestrogen-responsive element-independent oestrogen-like effects in oestrogen receptor-positive cells, providing insights into the tissue-selective oestrogenic effects of ICA. Additionally, ICA mimics oestrogen in the bones and brain, potentially reducing the risk of menopause-related skeletal and neurological disorders. However, ICA does not induce oestrogenic responses in the breast or uterus, indicating tissue-selective oestrogenic actions. The unique pharmacological properties of ICA include non-dose-dependent effects in vivo and regulating the circulating oestradiol and the dopamine metabolism [21].

Numerous autoimmune disorders have been shown to benefit from ICA treatment. Primary ovarian insufficiency is an autoimmune condition typically affecting the gynaecological system. A study investigated the effect of ICA in mice with autoimmune ovarian failure and its effect on immune regulation. The mice were injected hypodermically with three zona pellucida peptides to induce autoimmune primary ovarian insufficiency. The analysis methods included assessing the oestrous cycles, the FSH, LH, and AMH serum, and the anti-zona pellucida antibody (AZPAb) levels. In addition, flow cytometry was used to detect the expression of Th1 cells and Treg cells, and Western blotting was used to detect the expression of nuclear factor E2-related factor 2 (Nrf2), haemeoxygenase-1 (HO-1), and sirtuin-1 (Sirt1) proteins. Treatment with pZP3 decreased the serum AMH levels and increased the FSH, LH, and AZPAb levels. In addition, the number of healthy follicles decreased during all stages, while the number of atretic follicles increased. However, ICA reversed primary ovarian failure by upregulating Nrf2, HO-1, Sirt1, and Treg expression. The ICA treatment improved injured ovary structure and function in mice with autoimmune primary ovarian insufficiency. This mechanism was achieved by increasing the expression of the Nrf2/HO-1/Sirt1 pathway in the ovary and increasing the expression of Treg cells [44].

Previous research has demonstrated the preventive function of ICA against D-galactose-induced premature ovarian failure in mice. In another experimental study, D-galactose (200 mg/kg/day) and appropriate ICA doses (10, 50, and 100 mg/kg) for each group were administered to the experimental animals for 42 days. The levels of the following hormones in the serum were measured: anti-Müllerian hormone (AMH), LH, FSH, and oestradiol (E2). H&E-stained histological sections were used to evaluate the ovarian anatomy and follicle count. Western blotting was used to assess the preventive effects of ICA on cellular ageing and the DNA damage markers γH2AX and 53BP1 in primary ovarian granulosa cells. ICA downregulated the FSH and LH levels and upregulated oestradiol E2 and AMH, while promoting body and ovarian weight, follicle number, and fertility outcomes. It boosted cell survival, inhibited endogenous B-galactosidase activity, and shielded ovarian granulosa cells from D-galactose-induced ageing. Enhanced DNA damage repair was identified as the cause of the protective properties of ICA, as evidenced by the changes in the expression levels of the p53 binding protein 1 (53BP1) and γH2AX, a biomarker for DNA double-strand breaks. Hence, ICA can treat ovarian insufficiency early by reducing ovarian damage through the processes of repairing DNA damage [45].

The effects of ICA on ovarian function in a mouse model of D-galactose-induced ageing have been previously evaluated. The animals were treated with 200 mg/kg of D-galactose injected intraperitoneally for 50 days. ICA was administered at 50, 100, and 200 mg/kg per gavage between days 21 and 50. The evaluation methods included follicular histomorphology, serum hormone levels, female fertility examination, and ovarian mRNA and protein expression levels of AMH, Bax, and Bcl-2. The ICA treatment restored the serum FSH, LH, and E2 levels to their normal values in the mouse model employing old mice. Additionally, ICA improved female fertility by increasing the pregnancy rates, the average litter size, the birth weight, and the litter weight at weaning. The above study also analysed the ovarian expression levels of AMH, Bax, and Bcl-2 mRNA. The ICA-treated groups’ levels significantly improved compared to the control group [46].

A recent study evaluated the therapeutic potential of ICA in rats with polycystic ovary syndrome (PCOS) induced by letrozole and a high-fat diet. The ICA treatment significantly improved ovarian function and reproductive endocrine disorders by regulating sex hormones, restoring the oestrous cycle, and reducing ovarian morphological damage in the rats with PCOS. Additionally, reductions in weight gain and in the levels of triglycerides, fasting insulin, HOMA-IR, TNF-α, and interleukin-6 were observed with an increase in high-density lipoprotein cholesterol levels. Notably, ICA decreased apoptosis in the ovaries, supported by an increase in Bcl2 and a decrease in Bad and Bax. Furthermore, ICA was found to reduce the levels of p-JAK2/JAK2, p-STAT1/STAT1, p-STAT3/STAT3, and p-STAT5a/STAT5a, decrease the expression of IL-6 and gp130, increase the expression of the cytokine-inducible SH2-containing protein (CISH), and suppress cytokine signalling 1 (SOCS1). This study suggested that ICA could have a significant therapeutic effect in terms of improving polycystic ovary syndrome symptoms in rats by inhibiting IL-6/gp130/JAK2/STAT signalling and reducing ovarian apoptosis [47].

ICA regulates immunity and has antidepressant properties. Oestradiol levels are associated with menopausal depression. A prior investigation examined the application of ICA to the management of perimenopausal depression as a means of activating the PI3K/AKT pathway, a crucial mechanism involved in ovarian function and cell survival. In this study, researchers assessed the control of the immunological response and the hormone levels in a rat experimental paradigm. The animals were treated with ICA at 25 and 50 mg/kg doses for 30 days. The oestradiol E2, testosterone, and IL-2 levels increased with the ICA treatment, and the serum levels of FSH and LH decreased. Additionally, the expression of the oestrogen receptor by the hypothalamus and the concentrations of serotonin, dopamine, and norepinephrine in the brain increased. Moreover, ICA suppressed the expression of Bax and increased that of AKT, PI3K, phosphorylation-akt (p-AKT), and B-cell lymphoma 2 (Bcl-2). These results demonstrated that ICA treatment improved menopausal syndrome by regulating neurotransmitter production in the brain and rebalancing aberrant sex hormones in rats with perimenopausal depression via the PI3K-AKT pathway [48].

A previous study analysed the effect of ICA on lipopolysaccharide-induced endometritis in an experimental mice model. Endometritis was induced by an intrauterine infusion of 50 uL of LPS (1 mg/mL). After 24 h, 50 mg/kg ICA was administered intraperitoneally three times a day, six hours apart, over 36 h. The analysis methods included histopathological assays, wet-to-dry weight ratio calculations, myeloperoxidase activity assays, NO concentration determinations, ELISAs, the determination of oxidative stress markers, and RT-PCR analyses to measure the gene mRNA levels. The treatment with ICA significantly mitigated the uterine tissue injury caused by LPS, as observed through the histopathological examination. ICA administration also reduced the extent of inflammatory oedema, MPO activity, and NO production induced by LPS in the uterine tissue. In addition, ICA protected against LPS-induced inflammation and oxidative stress by changing the NF-κB and Nrf2 signalling pathways. The study suggested that ICA could be used as an anti-inflammatory treatment for conditions such as endometriosis, caused by *E. coli* [49].

The effects of ICA on immune tolerance in mice with recurrent spontaneous abortions have been examined using an experimental model of mice with an immune aetiology. The methods used in the study included administering ICA at a dose of 50 mg/kg/day to mice via gavage from day 0.5 to day 12.5 of gestation. The effects of ICA were assessed by analysing the abortion rates, conducting a histopathological analysis of the placentas, using flow cytometry to detect the lymphocyte populations, RT-PCR to measure the pro-inflammatory factors, ELISA to assess the cytokine levels, Western blotting to analyse protein expression, and flow cytometry to evaluate the Treg populations. The study also analysed the mTOR pathway using Western blotting to find mTOR-related proteins. The ICA treatment significantly reduced the abortion rate in animals with recurrent spontaneous abortions compared to the control group. Furthermore, the ICA treatment increased the ratio of labyrinth area to placenta in mice with recurrent spontaneous abortions, indicating a positive effect on placental development. Furthermore, the ICA treatment reduced the placental pro-inflammatory cells and factors, indicating an anti-inflammatory effect. Finally, the ICA treatment increased the population of regulatory T cells, which could have reduced the spontaneous abortion rate by improving immune tolerance [50].

Another study evaluated the effects of supplementation with Osthole and ICA on the productive performance of laying hens from 10 to up to 30 days. The methodology included evaluating the egg production rate, the egg quality, the body weight, the reproductive organ index, and the follicle count. With a dose of 2 mg/kg of each compound, the laying rate, the egg weight, the protein height, and the eggshell colour were significantly improved in the laying hens. The serum levels of reproductive hormones and gene expression in the ovaries were analysed. Techniques such as ELISA, RNA extraction, RT-qPCR, Western blotting, and HPLC were used. Combining Osthole with ICA significantly improved the laying rate, the weight, and the egg quality. The supplementation promoted the proliferation of granulosa cells in small yellow follicles and increased progesterone secretion. Furthermore, the study showed that Osthole combined with ICA increased the ER, FSHR, and PGR mRNA levels in the ovary, potentially improving ovarian development and ovulation. No Osthole and ICA residues were detected in the eggs [51].

**Table 2:** Summary of the main studies on the effect of ICA on female reproduction.

Type of Study	Model	Dose and Duration of Treatment	Main Findings	Refs.
In vivo In vitro	Sprague–Dawley female rats MCF-7, Ishikawa, SH-SY5Y, and MG-63 cells	50, 500, and 3000 mg/Kg, 3 months 10^−5^–10^−12^ M	ICA prevented oestrogen deficiency-induced osteoporotic changes in the bone structure, bone mineral density, and trabecular properties in the bone of ovariectomized rats. Moreover, it regulated the transcriptional events of the oestrogen-responsive genes related to bone remodelling and prevented the action of dopaminergic neurons against ovariectomized-induced changes. ICA exerted oestrogen-like activities and regulated the expression of oestrogen-responsive genes.	[21]
In vitro	Granulosa cells	5, 10, 25, 50, and 75 µg/L for 72 h	Ovarian granulosa cell proliferation and progesterone and oestrogen release were both markedly enhanced by ICA (10 μg/L). Furthermore, ICA increased CYP17 and CYP19 mRNA and protein expression.	[42]
In vitro	Mouse embryos	10, 20, 40, and 80 µM for 92.5 h	Decreased generation of reactive oxygen species and increased mitochondrial membrane potential were the two ways in which the ICA (40 µM) treatment fixed the negative effects of hydrogen peroxide on embryonic development.	[43]
In vivo In vitro	C57BL/6 female mice Ovarian granulosa cells	10, 50, and 100 mg/kg, 42 days 1, 10 μM, 6 h	ICA promoted ovary/body weight, follicle number, and fertility outcomes. Additionally, it downregulated the levels of the follicle-stimulating and luteinizing hormones and upregulated the levels of estradiol and the anti-Müllerian hormone. ICA repaired damaged DNA by changing the expression levels of 53BP1 and γH2AX, suggesting that it provided protection.	[45]
In vivo	Kunming white mice, female	50, 100, and 200 mg/kg	In a mouse model of D-galactose-induced ovarian ageing, icariin improved ovarian follicular development, inhibited follicular atresia, decreased the follicle-stimulating hormone and luteinizing hormone levels, increased estradiol expression, upregulated ovarian anti-Müllerian hormone expression, and increased the Bcl-2/Bax ratio.	[46]
In vivo	Male Sprague–Dawley rats	40 and 80 mg/kg, 30 days	ICA significantly improved ovarian function and reproductive endocrine disorders by regulating sex hormones, restoring the estrous cycle, and reducing ovarian morphological damage in rats with polycystic ovary syndrome. Additionally, ICA improved apoptosis in the ovaries.	[47]
In vivo	Sprague–Dawley female rats	12.5, 25, and 50 mg/kg, 30 days	ICA improved the pathological changes in the ovaries, elevated the serum levels of the female hormone estradiol, as well as testosterone and interleukin (IL)-2, decreased those of the follicle-stimulating hormone and the luteinizing hormone, promoted the expression levels of the oestrogen receptor (ER) and ERα in the hypothalamus, and increased those of serotonin, dopamine, and noradrenaline in the brain homogenate. Furthermore, ICA elevated the expression levels of AKT, phosphorylation-akt (p-AKT), PI3K (110 kDa), PI3K (85 kDa), and B-cell lymphoma 2 (Bcl-2) in the ovaries and inhibited those of Bax.	[48]
In vivo	Kunming female mice	50 mg/kg, three times a day, six hours apart	ICA inhibited the production of pro-inflammatory cytokines (IL-1ß, IL-6, and TNF-α) and boosted the production of anti-inflammatory cytokines (IL-10). Additionally, ICA modulated the LPS-induced expression of malondialdehyde, reactive oxygen species, superoxide dismutase 1, catalase, and glutathione peroxidase 1. Moreover, ICA improved the antioxidant defence system via the activation of the Nrf2 pathway.	[49]
In vivo	CBA/J female mice	50 mg/kg, 12 days	ICA decreased the number of recurrent spontaneous abortions. Furthermore, ICA reduced the expression of pro-inflammatory factors and was able to reduce the expression of a mechanical target of rapamycin (mTOR) in the placenta.	[50]
In vitro	Laying hens	2, 20, and 100 mg/kg, 30 days	ICA significantly improved the laying rate, weight, and egg quality parameters. Additionally, ICA increased the serum follicle-stimulating hormone, luteinizing hormone, and progesterone levels and granulosa cell proliferation.	[51]

The toxicity of ICA has not been completely documented. Recently, Wu et al. reported that the exposure of zebrafish embryos to ICA (10 and 40 µM) caused a reduction in the hatching rates and body length, in addition to an abnormal swim bladder. Furthermore, exposure to 40 µM ICA caused endocrine disruption of the thyroid due to an altered level of T4 and a significant decrease in the T3/T4 ratio. The authors also described an alteration in the transcription of the genes involved in thyroid development, thyroid hormone synthesis, and iodothyronine deiodinase. This is the first study reporting that ICA can cause adverse effects on embryonic development in a zebrafish model [52]. Additionally, the LD50 for ICA has not been reported in animal models, and safety data sheets indicate that no information has been provided thus far on its toxicity in specific organs or the negative effects of ICA. Therefore, toxicological research is necessary to determine the safety profile of ICA and the possible negative consequences that could occur from its use in humans or animals.

Limitations of the protective function of ICA for clinical application at the reproductive level require considering that the purity and concentration could vary after being isolated from different Epimedium species. Furthermore, its therapeutic application is limited by the lack of toxicological studies demonstrating toxicity and adverse effects, and the lack of research on ICA, specifically regarding its interactions with other drugs and its ability to increase pharmacological activity. The above reasons restrict the development of its therapeutic use.

## 4. Conclusions

As this article explains, various research studies have demonstrated that ICA can be used as a treatment for mammalian reproduction and that it enhances both male and female sexual function. It has beneficial effects on testicular morphology, sperm quality, erectile dysfunction, and reproductive dysfunction brought about by diabetes mellitus in men. The aforementioned effects occur through the elevation of the testosterone levels and the antioxidant capacity, the upregulation of gene expression for crucial reproductive function molecules like StAR and LRH, the activation of the ERα/Nrf2 pathways that govern cytoprotective antioxidant processes, and the stimulation of SIRT1-HIF-Iα, a crucial component of spermatogenesis and reproductive function. While ICA increases the biosynthesis of oestrogens and progesterone and the expression of the anti-Müllerian hormone, regulates the expression of the initiation factor eIF-1A, which is involved in embryonic implantation, and regulates various signalling pathways, it also inhibits the IL-6/gp130/JAK2/STAT, which is closely related to cell apoptosis, and activates the Nrf2 pathway, which activates the antioxidant defence system in female subjects. Additionally, in female subjects, ICA improves fertility, reduces the number of abortions, improves ovarian function, reduces the symptoms of polycystic ovary syndrome, and improves menopausal syndrome and the skeletal and neurological disorders that this causes. Further research is needed to determine how ICA affects other aspects of the fertilisation process, but, for now, the findings shown here demonstrate that ICA is advantageous for reproduction, suggesting its use as an alternative to treat reproductive problems in mammals.
